# Bacterial Population Changes during the Degradation Process of a Lactate (LA)-Enriched Biodegradable Polymer in River Water: LA-Cluster Preferable Bacterial Consortium

**DOI:** 10.3390/polym15204111

**Published:** 2023-10-17

**Authors:** Ryosuke Kadoya, Hitomi Soga, Miki Matsuda, Michio Sato, Seiichi Taguchi

**Affiliations:** 1 Department of Food and Nutrition, School of Life Studies, Sugiyama Jogakuen University, 17-3 Hoshigaoka Motomachi, Chikusa-ku, Nagoya 464-8662, Aichi, Japan; hichanyamibani@gmail.com (H.S.); disw.mk86@gmail.com (M.M.); 2 Microbial Genetics Laboratory, Department of Agricultural Chemistry, Graduate School of Agriculture, Meiji University, 1-1-1 Higashimita, Tama-ku, Kawsaki 214-8571, Kanagawa, Japan; mittyan@meiji.ac.jp; 3 Graduate School of Science, Technology and Innovation, Kobe University, 1-1 Rokkodai-cho, Nada, Kobe 657-8501, Hyogo, Japan; staguchi86@people.kobe-u.ac.jp

**Keywords:** LAHB [poly(lactate-*co*-3hydroxybutyrate)], biodegradable polymer, river environment, microorganisms, bacterial diversity

## Abstract

The lactate-based polyester poly[lactate (LA)-*co*-3-hydroxybutyrate (3HB)], termed LAHB, is a highly transparent and flexible bio-based polymeric material. There are many unknowns regarding its degradation process in riverine environments, especially the changes in bacterial flora that might result from its degradation and the identities of any LAHB-degrading bacteria. LAHB were immersed in the river water samples (A and B), and LAHB degradation was observed in terms of the weight change of the polymer and the microscopic changes on the polymer surfaces. A metagenomic analysis of microorganisms was conducted to determine the effect of LAHB degradation on the aquatic environment. The bacterial flora obtained from beta diversity analysis differed between the two river samples. The river A water sample showed the simultaneous degradation of LA and 3HB even though the copolymer was LA-enriched, suggesting preferable hydrolysis of the LA-enriched segments. In contrast, only 3HB degraded for the LAHB in the river B water sample. The linear discriminant analysis effect size (LEfSe) analysis revealed 14 bacteria that were significantly increased in the river A water sample during LAHB degradation, suggesting that these bacteria preferentially degraded and assimilated LA-clustering polymers. Our metagenomic analysis provides useful insights into the dynamic changes in microbial communities and LA-clustering polymer-degrading bacteria.

## 1. Introduction

Eight million tons of plastic waste are dumped into aquatic environments each year. It is estimated that 150 million tons are already in the ocean, and this amount is increasing every year [1]. Plastic is a convenient material because it is lightweight, easy to process, water-resistant, and inexpensive. When plastic waste is dumped into the ocean, it drifts in the water for a long time. There have been problems with marine organisms mistaking plastic for food and eating it, resulting in their deaths [2]. Another issue that has become a hot topic in recent years is the presence of microplastics in the ocean [3]. Microplastics are fine plastic particles with diameters of up to 5 mm. Microplastics include plastic bottles and other plastic products dumped into the aquatic environment that have been crushed by waves or broken down into smaller pieces by ultraviolet light [4,5]. Recently, some abrasives (plastics) in toothpaste have been washed out in aquatic environments. Microplastics adsorb toxic components and are ingested by marine organisms, and they are believed to adversely affect their health [6,7].

One of the solutions to these problems caused by plastic pollution is making biodegradable bioplastics. Biodegradable bioplastics are required to have the same physical properties as conventional petroleum-based plastics, but they are also able to completely decompose in water and via carbon dioxide in the environment [8,9]. Polylactic acid (PLA), a representative bioplastic, is thermoplastic, has excellent transparency, and can be processed into containers such as polyethylene terephthalate (PET) bottles [10]. PLA has attracted attention as an important material for addressing the problems of oil depletion, greenhouse gas reduction, and microplastics. One serious problem is that PLA is nondegradable in water environments such as freshwater and sea water [11]. In contrast, poly(3-hydroxybutanoic acid) [P(3HB)] is biosynthesized for carbon storage in microorganisms, and it has degraded in the environment [12]. Many microorganisms synthesize and accumulate high-molecular-weight polymers in their cells under conditions of nutrient source (such as nitrogen and phosphorus) depletion [13].

In 2008, for the first time, we succeeded in producing LAHB using recombinant *Escherichia coli* and our newly developed lactate (LA)-polymerizing enzyme (LPE) [14]. LAHB exhibits distinct properties compared with those of PLA and P(3HB) homopolymers. These homopolymers are rigid, whereas LAHB is flexible and stretchable depending on the LA fraction [15]. For example, P(30 mol% LA-*co*-3HB) [30% LAHB] exhibits thermal properties similar to those of petroleum-derived polypropylene. Recent advances in microbial LAHB production platforms, achieved through metabolic engineering, have provided enough LAHB for various polymer analyses, now reaching levels even higher than that of P(3HB) [16,17]. These results suggest that LAHB could be used in a wide range of applications. Furthermore, LAHB has proven effective in addressing various environmental problems caused by plastics [18]. In this sense, LAHB is an evolved version of PLA.

To date, the molecular mechanisms of polymer degradation have been studied using extracellular degrading enzymes produced by microorganisms isolated from various environments [19]. Bacteria and fungi produce extracellular depolymerases that can degrade polymers under anaerobic and aerobic conditions [20]. The enzymatic degradation of a polymer surface produces monomers and oligomers. Oligomers are hydrolyzed via intracellular hydrolases to form monomers. Finally, the monomer is converted into an energy source for the microorganism [21,22].

There have been reports on the microbial ecology that is associated with the biodegradation of LAHB in soil but not in water [23]. In this study, we focused on the impact of LAHB degradation on the microbial community in the river water samples based on metagenomic analysis. We investigated how the microbial community changed after LAHB was introduced into the river water samples collected from the two sites. Metagenomic analysis revealed that one river harbors bacteria that specifically degrade the LA-clustering region within the LA-enriched LAHB. These results indicate that there are bacteria in water that efficiently degrade LA-clustering region polymers, which are considered persistent. We also believe that clarifying the changes in the environmental microbial community brought about by LAHB degradation will be an important milestone for realizing the industrialization of LAHB.

## 2. Materials and Methods

### 2.1. Preparation of Several Polymers

The expression vector pTV118N*pctphaC1*_Ps_(ST/QK)*AB*, which harbors genes encoding propionyl-CoA transferase from *Megasphaera elsdenii* (*pct*), engineered PHA synthase with LA-polymerizing activity [*phaC1*_Ps_(ST/QK)] from *Pseudomonas* sp. 61-3 and the 3HB-CoA supplying enzymes, β-ketothiolase and acetoacetyl-CoA reductase (*phaA*, and *phaB*) from *Ralstonia eutropha*. This plasmid was used for LAHB production. In addition, recombinant cells harboring pGEM*phaC1*_Ps_(ST/QK)*AB* were used for P(3HB) production. For polymer production, the recombinant *E. coli* strain, BW25113, harboring pTV118N*pctphaC1*_Ps_(ST/QK)*AB* or pGEM*phaC1*_Ps_(ST/QK)*AB*, were grown on a 100 mL LB medium containing 20 g/L glucose at 30 °C for 48 h with reciprocal shaking at 120 rpm. Ampicillin (Amp; 100 μg/mL), was added when needed.

### 2.2. HPLC Analysis of Polymer

After cultivation, the *E. coli* was harvested via centrifugation at 1690× *g* for 15 min. The polymer content was determined as described previously [16]. Cells were frozen overnight at −80 °C. The lyophilized cells were treated with 1 mL of concentrated sulfuric acid at 120 °C for 45 min to convert the polymer to unsaturated carbonate. The sample was cooled to room temperature, diluted 10-fold with 0.014 N sulfuric acid (0.007 mol/L), and vortexed vigorously. The samples were centrifuged at 4 °C and 10,000× *g* for 15 min. The supernatant was transferred to another tube and used as the HPLC sample. The sample was quantified using HPLC (LC-2030C, SHIMAZU, Kyoto, Japan), equipped with a UV detector at 210 nm, and an aminex HPX-87H ion exclusion column (7.8 mm I.D × 300 mm, Bio-Rad laboratories, Hercules, CA, USA). The buffer (0.014 N sulfuric acid) flow and temperature conditions during the measurements were as follows: the flow was 0.6 mL/min, and the column oven was heated at 60 °C. LA and 3HB were measured and the LA content in the polymer was calculated from the standard curve.

### 2.3. Growth Conditions of Bacteria from the River Water Sample

**Figure 1:**
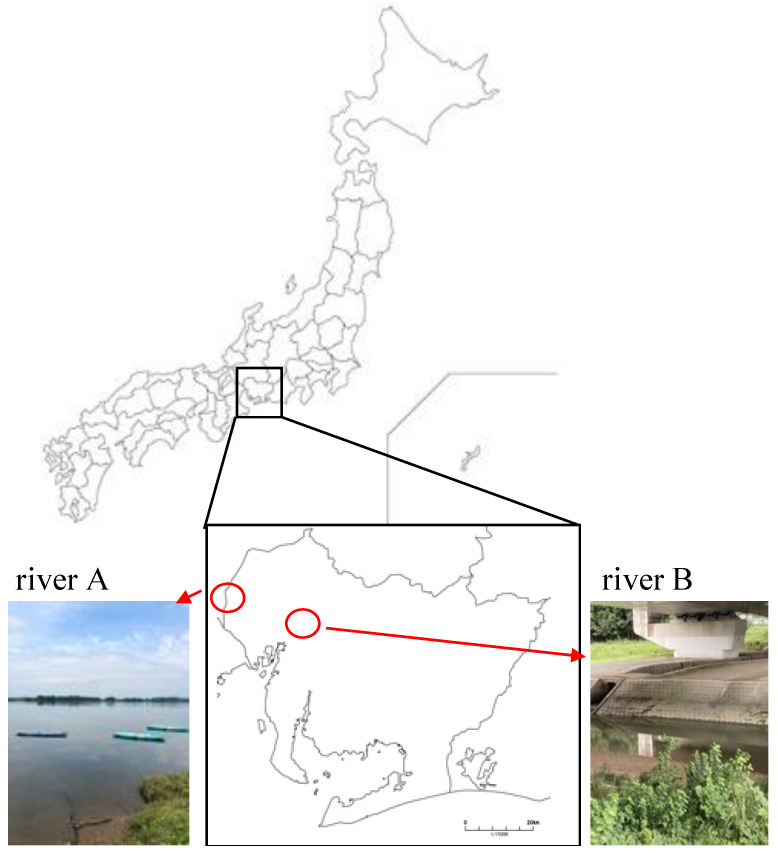
Locations and photos where river water samples were taken. The samples were collected from two rivers in the Aichi Prefecture, Japan. Red circles in the figure indicate river locations. River A is 35°07′28.3″ N 136°58′44.9″ E and River B is 35°15′10.0″ N 136°41′42.8″ E.

River water samples were collected at 35°07′28.3″ N 136°58′44.9″ E (river A) and 35°15′10.0″ N 136°41′42.8″ E (river B). They are rivers in the Aichi prefecture, and the samples were taken on 3 July and 10 July 2021 (Figure 1).

To assess the biodegradability of the polymer in river water, four types of polymer films, 28% LAHB, 69% LAHB, P(3HB), and polyethylene (PE), were used. A polymer film (1 × 2 cm) was placed in a 20 mL river water sample and incubated statically at 25 °C in a 30 mL test glass tube. In the analysis of the microbial community, each polymer film (28% LAHB, 69% LAHB and P(3HB)) was used as a carbon source.

### 2.4. Measurement Weight and LA Content of LAHB after Immersion in the River Water Samples

To determine the weight and LA content of LAHB after immersion in the river water samples, the following methods were used. After immersion in river water, all polymers were collected from test glass tubes and dried in a vacuum for 1 day to remove water. After measuring the dry weight of each polymer sample, each polymer was dissolved in concentrated sulfuric acid and used as a sample for HPLC.

### 2.5. Measurement of Cell Density Using Flow Cytometry

The volumetric cell density (cells/mL) was measured via flow cytometry equipped with a SH800 cell sorter (SONY, Tokyo, Japan). Cells grown under the aforementioned conditions were harvested, and a 10-fold diluted sample with water was used for cell counting. Bacterial cells were stained with DAPI (Dojindo Laboratories, Kumamoto, Japan); 1 μL of DAPI solution was added to 1 mL of cell suspension and mixed, then, the cells were incubated at room temperature for 5 min. The flow rate of flow cytometry was set to 11 μL/min. Samples were measured for the number of DAPI-stained particles per minute. All data for FSC (forward scatter) and SSC (side scatter) images were recorded using SH800 software Ver 1.8 (SONY, Japan).

### 2.6. Scanning Electron Microscope

The change in morphology of each polymer film 28% LAHB, 69% LAHB, and P(3HB) in the biodegradation experiments was observed using a scanning electron microscope (SEM, JSM-6700F JEOL, Tokyo, Japan). The samples were mounted on brass specimenholder and coated with osmium using HPC-1 (Vacuum Device Co., Ibaraki, Japan) ion sputter prior to SEM observation.

### 2.7. Preparation of Samples for Metagenome Analysis and Metagenome Analysis Using NGS

Bacterial cells were collected from 15 mL of river water via centrifuge (10,000× *g*, 20 min, 4 °C). Genomic DNA was extracted from the pellets using the DNeasy^®^ PowerBiofilm kit (Qiagen, Hilden, Germany), in accordance with the manufacturer’s instructions. Finally, the extracted DNA was eluted in autoclaved distilled water, and the yield was measured using a NanoDropTM 2000 spectrophotometer (Thermo Scientific, Waltham, MA, USA).

Genomic DNA was used for profiling the river water sample microbial community through 16S rRNA amplicon sequencing on the Illumina MiSeq platform. Primers 314F (5′-ACACTCTTTCCCTACACGACGCTCTTCCGATCT-NNNNN–CCTACGGGNGGCWGCAG-3′) and 805R (5′-GTGACTGGAGTTCAGACGTGTGCTCTTCCGATCT-NNNNN–GACTACHVGGGTATCTAATCC-3′) were used to amplify the V3–V4 hypervariable regions of the bacterial 16S rRNA gene using PCR on a thermocycler (Takara PCR Cycler Dice Gradient, Takara, Japan). PCR was conducted using the following program: initial denaturation at 95 °C for 3 min, 27 cycles of denaturation at 95 °C for 30 s, annealing at 55 °C for 30 s, elongation at 72 °C for 60 s, and a final extension at 72 °C for 10 min. A PCR reagent was used (TaKaRa Ex Taq^®^ Hot Start Version (Takara, Kusatsu, Japan)). Sequencing libraries were normalized for DNA concentration, pooled, then sequenced on an Illumina MiSeq for 300 bp × 2 paired end reads with the V3–V4 kit.

### 2.8. Microbial Community Analysis

Microbial community analysis was performed using the QIIMEII software package version 2021.11, as follows [24]. The demultiplexed paired-end sequencing protocol was used to import the sequences, and the dada2 denoise-paired command was used to delete low-quality sequences. The sequences were grouped into operational taxonomic units (OTUs) with 97% similarity.

Alpha diversity measures the richness of species in a given community. Beta diversity, on the other hand, analyzes different compositions, in terms of abundance of different taxa, between different samples. The Chao1 and Shannon Index analysis of the alpha diversity was performed. The beta diversity analysis was conducted by calculating the unweighted UniFrac distance between each pair of samples. The taxonomic assignment used a classification based on a filtrate of the OTUs sequence, from the SILVA SSU database release 138 to the V3–V4 region. The sequences obtained were filtered and assigned to at least one genus.

We analyzed differential microbial abundances between the two groups with Linear discriminant analysis Effect Size (LEfSe) in the Galaxy web application (http://huttenhower.sph.harvard.edu/galaxy (accessed on 16 June 2022)) [25]. Based on the LEfSe results, with a threshold of log10 LDA > 2, a cladogram was plotted.

## 3. Results and Discussion

### 3.1. Microbial Degradation of Polymers Containing LA in River Waters

P(3HB) (which is reportedly biodegraded well by microorganisms in natural environments), LAHB (a random copolymer of LA and 3HB), and polyethylene (PE) (which is not degraded in the environment) were prepared. LAHBs with different LA contents (28 mol% LAHB and 69 mol% LAHB (28% LAHB and 69% LAHB)) were prepared in this study. LAHB with a high LA content has many long regions (LA-clustering regions) where LA polymerizes with itself in the polymer. Previous LAHB degradation studies have confirmed that LAHB and LA-clustering regions were degraded by microorganisms in the soil [21]. However, the degradation of LAHB and LA-clustering regions in river water has not been confirmed. Therefore, we immersed LAHB in the river water samples from two different locations and observed degradation.

**Figure 2:**
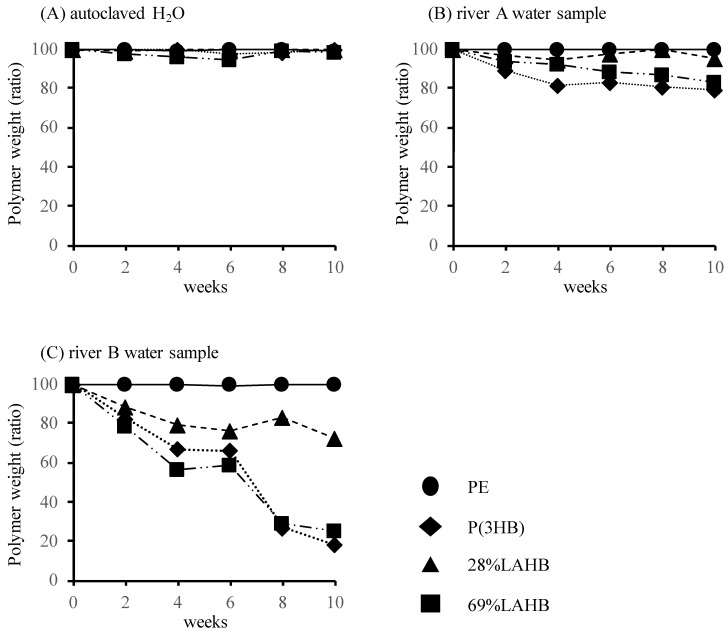
The weight loss of LAHB and P(3HB) after immersion in each river water sample. (**A**) Autoclaved H_2_O, (**B**) river A water sample, (**C**) river B water sample. Black circle: weight of polyethylene sample. Black diamond: weight of P(3HB) sample. Black triangle: weight of 28% LAHB sample. Black square: weight of 69% LAHB sample.

Figure 2 shows the time course of the weight change of each polymer film in the river water samples from two different locations. The weight of the polymer decreased over time in both river water samples, but no weight loss was observed in the autoclaved water, including ‘not-living’ microorganisms (Figure 2A). Therefore, the reduction in the weight of these polymers was inferred to be due to the biodegradation of the polymers which occurred because of the microorganisms in the river water samples. There was a significant difference in the weight change of the polymer between the two river water samples. In the river A water sample, the weight loss of each polymer was approximately 20% after 10 weeks (Figure 2B). In the river B water sample, P(3HB) and 69% LAHB exhibited 80% weight loss (Figure 2C). We attributed the difference in weight loss to the different numbers of microorganisms and microbial communities in the river. The number of microorganisms in each river was measured to be 0.6 × 10^7^ cells/mL in the river A water sample, and 1.2 × 10^7^ cells/mL in the river B water sample.

**Figure 3:**
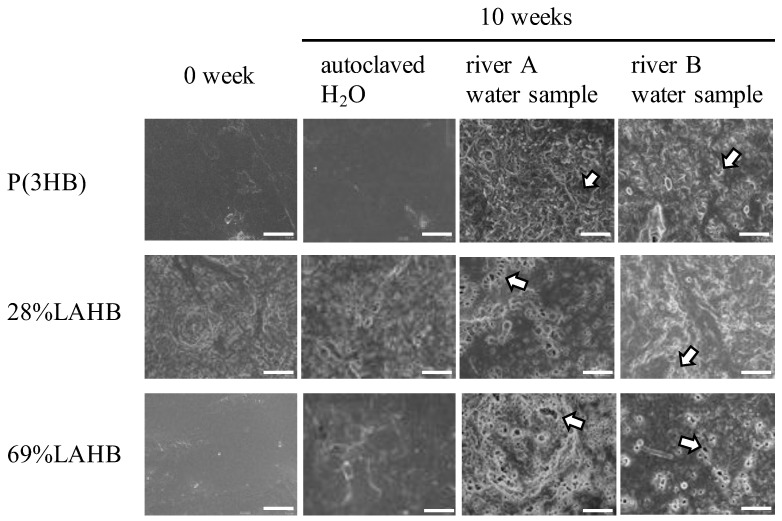
Transmission electron microscopic images of the surface of P(3HB) and LAHB. It shows the surface of P(3HB) and LAHB after 0 and 10 weeks of immersion in the river water and autoclaved H_2_O. The white bar in the photo represents 5 μm. The surface of P(3HB) and LAHB showed the presence of holes (holes indicated by the white arrows).

Electron microscopy showed signs of degradation on the surface of each polymer (P(3HB), 28% LAHB, and 69% LAHB) (Figure 3). The surface conditions of each polymer immersed in autoclaved water remained unchanged for 10 weeks. Holes were identified on the surface of the polymer immersed in both river water samples for 10 weeks (as shown in Figure 3, where the holes are indicated by white arrows). These holes were not observed before immersion in each river water sample. The formation of holes on the polymer surface were assumed to be due to microbial degradation.

### 3.2. Statistical Evaluation of Bacterial Diversity in the River Water Samples under LAHB Degradation

**Figure 4:**
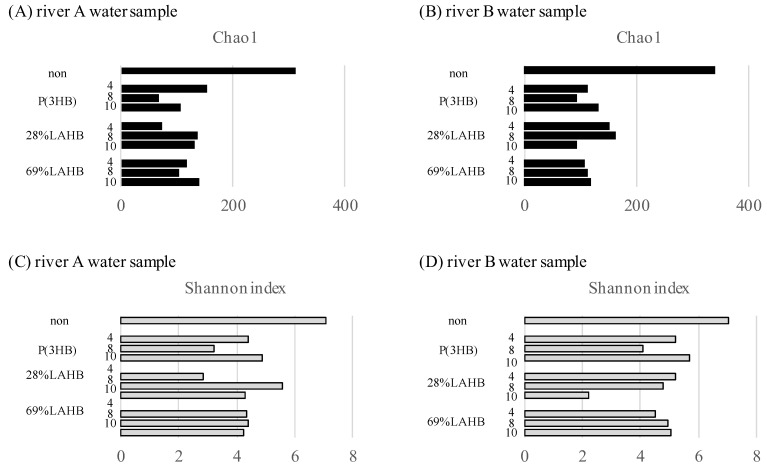
Alpha diversity of microbial communities in the river water samples with and without polymer degradation. (**A**) Chao1 index of the river A water sample, (**B**) Chao1 index of the river B water sample, (**C**) Shannon’s index of the river A water sample, and (**D**) Shannon’s index of the river B water sample.

The effect of the degradation of each polymer (28% LAHB, 69% LAHB, and P(3HB)) on bacterial diversity in the river water samples was statistically examined using alpha and beta diversities from metagenomic data. As shown in Figure 4, the alpha diversity (Chao1 and Shannon index) of each river water sample (A and B) was lower than before polymer degradation. These results suggest that the degradation of each polymer reduced the bacterial diversity of the river water samples. The diversity of the microbial community did not change with degradation or over time (Figure 4 shows no change after 4 and 10 weeks). This result indicated that the experimental system comprising the test tubes was a closed system.

**Figure 5:**
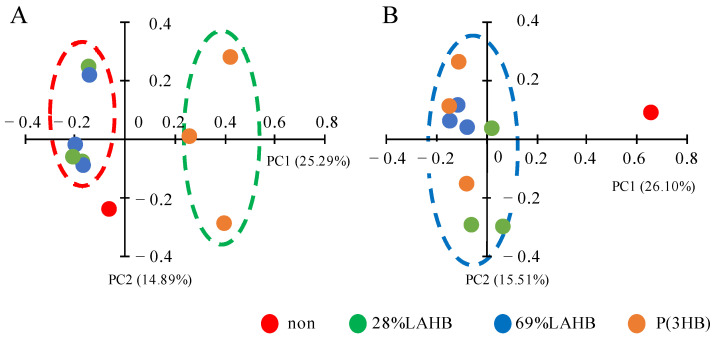
Plot of PCoA using unweighted UnuniFrac distances showing the beta diversity of microbial communities among river samples with and without polymer degradation. Each point represents an individual sample. Red circle: polymer not immersed. Green circle: immersed in 28% LAHB. Blue circle: immersed in 69% LAHB. Red circle: immersed in P(3HB). Ellipses represent clusters. (**A**) PCoA of the river A water sample. PC1 explained 25.29% of the variation observed, and PC2 explained 14.89% of the variation observed. (**B**) PCoA of the river B water sample. PC1 explained 26.10% of the variation observed, and PC2 explained 15.51% of the variation observed.

Regarding beta diversity calculated by unweighted UniFrac distances, the results of the principal coordinate analysis (PCoA) indicated that the microbial community in each river sample was significantly altered by polymer degradation (Figure 5). In the river B water sample, the microbial communities formed the same clusters during LAHB (28% and 69%) and P(3HB) degradation (Figure 5B, blue ellipse). The P(3HB)-degrading and LAHB-degrading microbial communities formed distinct clusters in the river A water samples (Figure 5A, red ellipse: cluster of LAHB degradation. Green ellipse: cluster of P(3HB) degradation). During LAHB degradation in the river A water sample, the degradation and metabolism of the LA-clustering region polymers were thought to impose a selective pressure on a different group of microorganisms compared with what occurred during P(3HB) degradation. Interestingly, the microbial community in the same river showed different changes depending on the type of polymer being degraded. In the river A water sample, LAHB was degraded to produce both LA and 3HB, whereas P(3HB) was degraded to produce 3HB. The two clusters in the river A water sample were thought to be formed by different degradation products. On the other hand, LAHB only produced 3HB when it was degraded by the microorganisms in the river B water sample. Therefore, it was considered that the same clusters were formed during P(3HB) degradation.

**Figure 6:**
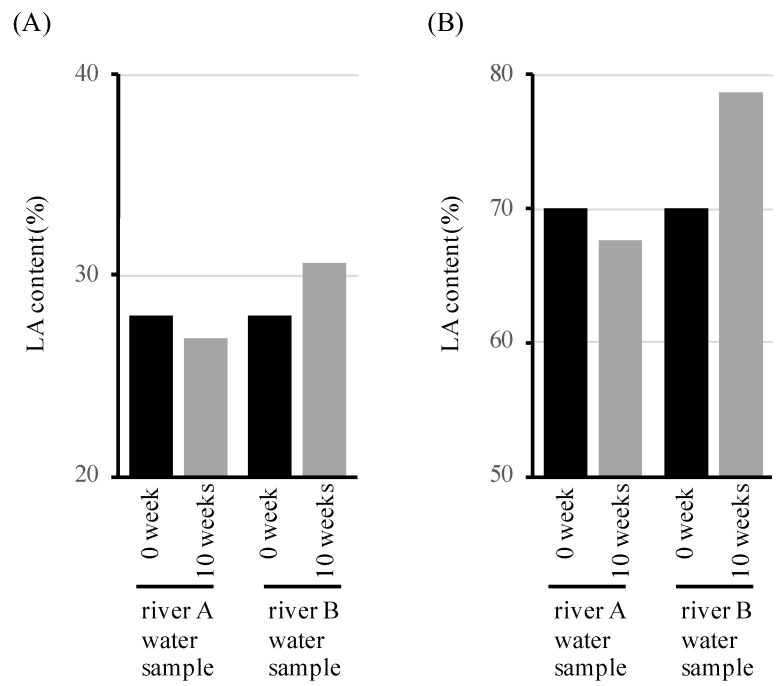
LA content of LAHB. It shows the LA content of LAHB film at 0 and 10 weeks after immersion in the river water samples. (**A**) LA content of 28% LAHB before and after 10 weeks of immersion in the river water samples. (**B**) LA content of 69% LAHB before and after 10 weeks of immersion in the river water samples.

If the microbial community in the river water sample degraded and assimilated the LA-clustering and 3HB-clustaring regions, the LA content in the polymer should not change. On the other hand, if the 3HB-clustaring region is degraded and assimilated, and the LA-clustering region cannot be degraded and assimilated, the LA content of the polymer should increase. Therefore, the LA content of the undegraded polymer of the river water samples was assessed using high-performance liquid chromatography (HPLC) after 10 weeks of immersion in the river water samples. Moreover, 28% LAHB and 69% LAHB were immersed in the river A water sample, and there was no change in the LA content of the LAHB after 10 weeks (Figure 6A,B). However, when immersed in the river B water sample for 10 weeks, the LA content in the polymer increased. The LA content increased to 31% at 28% LAHB, and to 79% at 69% LAHB, after 10 weeks. In the river A water sample, it is possible that the 3HB-clustering region and the LA-clustering region in the LAHB were simultaneously degraded by microorganisms. In the river B water sample, only the 3HB cluster region in LAHB was considered to be degraded by microorganisms. These results suggest that there were microorganisms in the river A water sample that degraded the LA-clustering region.

### 3.3. Characterization of the Microbial Community in the River Water Samples under LAHB Degradation Conditions

**Figure 7:**
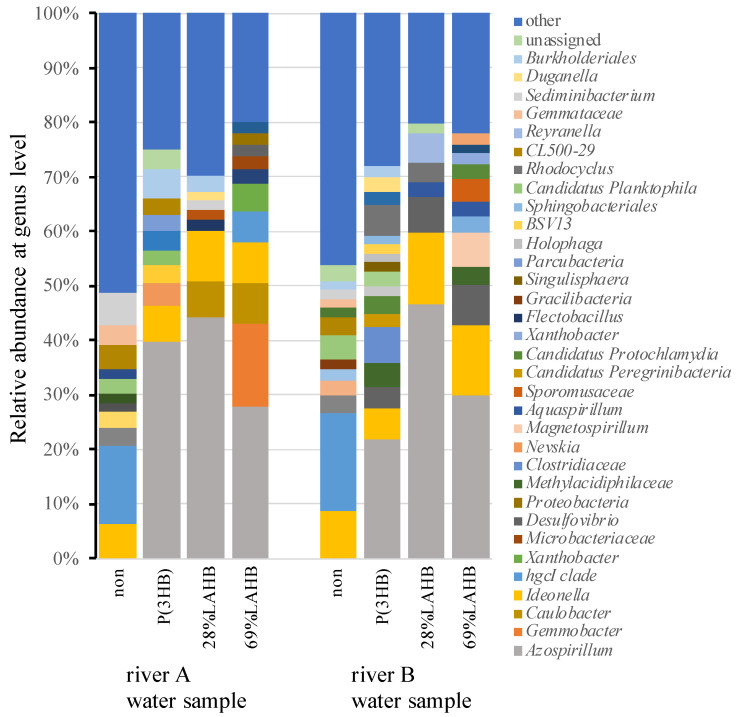
Relative abundance of the river water sample bacterial communities at the genus level for each polymer degradation. Each color represents the relative abundance of a bacterial taxon on the stacked bar chart.

Polymer degradation by microorganisms has been reported to be performed by extracellularly secreted PHB depolymerase [26,27]. In such an environment, polymers are considered to be one of the carbon sources that microorganisms degrade and use for metabolism-related purposes. Several studies have reported changes in the microbial community when P(3HB) is used as the carbon source in river water [28]. The percentage of P(3HB)-degrading bacteria in the environment is known to be 0.5–9.6% of the total [28]. These P(3HB)-degrading bacteria belong to the genera *Acidovorax* [29], *Ideonella* [30], *Azospirillum* [31], and *Variovorax* [22,32]. The degradation of P(3HB) has been suggested to affect the growth of microorganisms in the environment [28]. It is unknown how LAHB, a novel polymer, affects the microbial community when degraded in river water. Figure 7 shows the changes in the microbial community after the degradation of LAHB and P(3HB) in the river A and river B water samples. A significant increase in the percentage of *Azospirillum* was observed under all polymer degradation conditions.

**Figure 8:**
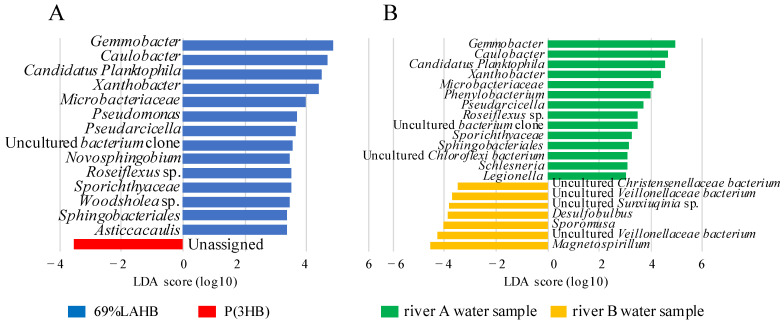
Linear discriminant analysis (LDA) demonstrated distinct bacterial genera that increased due to LAHB degradation. Genera with *p* < 0.05 and an LDA score > 2 were considered significant and are shown here. (**A**) Compared the microbial community between 69% LAHB and P(3HB) degradation in the river A water samples using the LEfSe analysis. (**B**) Compare the microbial community for 69% LAHB degradation in the river A and B water samples using LEfSe analysis.

In the beta diversity analysis (Figure 5), the LAHB- and P(3HB)-degrading microbial communities formed different clusters in the river A water sample, whereas they formed the same cluster in the river B water sample. Therefore, we compared the microbial community between 69% LAHB and P(3HB) degradation in the river A water samples using the linear discriminant analysis effect size (LEfSe) approach (Figure 8A). The results showed that bacteria of 14 genera increased specifically during LAHB degradation. Among these microorganisms, genera such as *Gemmobacter* sp. [33], *Caulobacter* sp. [34], *Candidatus Planktophila* [35], *Xanthobacter* sp. [36], *Microbacteriaceae* [37], *Pseudomonas* [38], and *Novosphingobium* sp. [39] have been documented as possessing homologous genes encoding PHB depolymerases. It is possible that the depolymerase of these bacteria degrades the LA-clustering region. *Asticcacaulis* possesses a homologous gene for PHB depolymerase; however, it is intracellular [40]. The other bacterial genera could have increased due to the assimilation of LAHB degradation products. In addition, the microbial communities degrading 69% LAHB in the river A and river B water samples were compared in LEfSe (Figure 8B). The results revealed a significant increase in 14 bacterial genera within the water sample from river A. These 14 genera included bacteria from 5 genera that harbored the homologous gene for PHB depolymerase, as shown in Figure 8A. The microbial community in the river A water samples with LAHB immersion may have been affected by its own capacity for LA-clustering region degradation and the assimilation of LA. These results were obtained using bioinformatics analysis. Therefore, it is necessary to isolate the LA-clustering region degrading bacteria from the river A water samples during LAHB degradation. It is a very important experiment to directly determine the mechanism of microbial LAHB degradation as indicated by bioinformatics. Previous studies have shown that the degrading enzyme of *Variovorax* sp. C34, extracted from soil, is able to recognize LA trimer and cleave the LA-LA bond [22]. To understand the mechanism of LAHB degradation via the river water microorganisms identified in this study, the microorganisms must be isolated from the river water A samples and the enzymes that are to be purified. We hope to address this issue in the next step. The findings obtained here would allow us to think about the factors affecting the potential biodegradability of PLA in the environments including riverine sites.

## 4. Conclusions

In this study, the effects of LAHB degradation on the diversity of microbial communities in different river water samples were characterized using a metagenomic analysis. The results suggest that the mode of microbial community convergence is dynamically changed by the degradation of LAHB, as well as the degradation of P(3HB). This study identified bacterial species that increased in number only during LAHB degradation in the river A water sample. This dominant effect was caused by the degradation and assimilation of LA-clustering regions by certain bacteria. In future studies, more natural environments would be good targets for the investigation of the additional effects of LAHB using metagenomic analysis. In addition, we will identify LA-clustering regions that degrade microorganisms, as well as their degradation mechanisms. Consequently, we will address the factor(s) that affect the degradation of PLA, a representative and well-studied biobased polymer with a wide range of potential applications.

## Data Availability

All data included in this study are available upon request by contact with the corresponding author.
